# Doctor-Shopping Behavior among Patients with Eye Floaters

**DOI:** 10.3390/ijerph120707949

**Published:** 2015-07-13

**Authors:** Gow-Lieng Tseng, Cheng-Yu Chen

**Affiliations:** 1 Department of Ophthalmology, Taipei Municipal Hospital, Renai Branch, No.10, Sec. 4, Ren’Ai Rd., Taipei 10629, Taiwan; 2 Department of Health Promotion and Health Education, National Taiwan Normal University, No. 162, Sec.1, Heping East Road, Taipei 10610, Taiwan; E-Mail: t09004@ntnu.edu.tw

**Keywords:** myodesopsia, eye floaters, doctor-shopping behavior, motivation

## Abstract

Patients suffering from eye floaters often resort to consulting more than one ophthalmologist. The purpose of this study, using the Health Belief Model (HBM), was to identify the factors that influence doctor-shopping behavior among patients with eye floaters. In this cross-sectional survey, 175 outpatients who presented floaters symptoms were enrolled. Data from 143 patients (77 first time visitors and 66 doctor-shoppers) who completed the questionnaire were analyzed. Descriptive and logistic regression analyses were performed. We found that women and non-myopia patients were significantly related with frequent attendance and doctor switching. Though the HBM has performed well in a number of health behaviors studies, but most of the conceptual constructors of HBM did not show significant differences between the first time visitors and true doctor-shoppers in this study. Motivation was the only significant category affecting doctor-shopping behavior of patients with eye floaters.

## 1. Introduction

Myodesopsia (eye floaters) is a common complaint in ophthalmologic clinics. Most patients described the floaters as spots, threads, hair-like, hollow circles, or cobwebs, with or without flashes. These symptoms are usually secondary to degenerative changes (liquefaction) in the vitreous body. The liquefied vitreous fluid may leak out from the vitreous body and make posterior vitreous surface separated from retina, leading to posterior vitreous detachment (PVD). The prevalence of eye floaters in the general population increase from 24% in adults aged 50 to 59 years to 87% among those aged 80 to 89 years [1]. In 14% cases, myodesopsia is associated with a retinal tear and if not treated properly 33% to 46% of them may lead to retinal detachment [1,2]. 

Patients may consult an ophthalmologist when experiencing such symptoms for the first time. But since the symptoms will persist for a period of time and sometimes become annoying, patients may consult the same or different ophthalmologists without referral, over and over again. It is not only the wastage of medical resources but also burden of medical services employees. This “doctor-shopping” phenomenon, implicating frequent attendances and switching of physicians, has been reported in Taiwan [3], Japan[4,5], Hong Kong [6,7], and other countries [8]. These studies focused on general medicine or pediatric clinics; the psychological background of the behavior was seldom discussed. There was a qualitative study describing the psychological perspective of a small number of patients with eye floaters, but the issue of doctor-shopping was not discussed [9]. 

Implicit conceptualization of the health belief model (HBM) is the combination of perceived susceptibility with perceived severity (perceived threat), and perceived benefits with perceived barriers (evaluation of the course of action taken). As such, health behavior is more likely to be carried out if the individual perceives threat of disease (*i.e.*, high susceptibility and severity), if benefits can be derived from performing the behavior, if there are few barriers to performing the behavior, or some combination of these. Similarly, if one is “motivated”, behavioral action is more likely [10]. 

Health Belief Model (HBM) has been applied in health behaviors studies, including: (a) preventive health behaviors, which include health-promoting (e.g., diet, exercise) and health-risk (e.g., smoking) behaviors as well as vaccination and contraceptive practices; (b) sick role behaviors, which refer to compliance with recommended medical regimens, usually following professional diagnosis of illness; and (c) clinic use, which includes physician visits for a variety of reasons [11]. 

There are seven key concepts in HBM: (1) perceived susceptibility (beliefs about the likelihood of getting a disease or condition); (2) perceived severity (a person’s feelings about the seriousness of contracting an illness or of leaving it untreated including evaluations of both medical and clinical consequences and possible social consequences; (3) perceived benefits (the opinion of the effectiveness of advised action to reduce the risks of a condition); (4) perceived barriers (one’s beliefs regarding the total costs of implementing the recommended action); (5) self-efficacy (the conviction that one can successfully execute the behavior required to produce the outcomes); (6) motivation related to performing the health behaviors; and (7) cues to action [12,13,14]. The model’s basic assumption is that once an individual is aware of a health risk, an assessment of costs and benefits, coupled with cues, motivates action. 

In this study, we used the framework of HBM to examine the relationship between its conceptual variables and doctor-shopping behavior among patients with eye floaters, focusing on the relationship between illness perceptions and utilization of health services. 

## 2. Methods

### 2.1. Sample and Data Collection

We enrolled consecutive patients presenting primarily symptoms of eye floaters at the department of ophthalmology, Taipei Municipal Hospital, Renai Branch, Taiwan, from 8 April 2013 to 31 May 2013. Patients were eligible to participate in this study if they met the following criteria: (1) 18 years old or above, (2) presenting symptoms of floaters in one or both eyes less than 6 months, and (3) no past history of cataract or vitreoretinal surgery. 

### 2.2. Measures

All of the enrolled patients signed an informed consent and completed a self-administered questionnaire while waiting for eye examinations. The validity of the questionnaire have been established by several experts and the content validity index of all items were from 0.7 to 1.0. The reliability of the questionnaire have been established by pretest in 30 patients with eye floaters, and internal consistency Cronbach’s alpha value was 0.8. The whole document was approved by the institutional review board of Taipei Municipal Hospital on 8 April 2013 (ID code: TCHIRB -1020207-E). 

The questionnaire consists of two parts. The first part collects socio-demographic information, include age, sex, education, family monthly income, myopia status, eye floaters characters, consultation times, and reasons. The second part measures the HBM variables, including the following seven categories: susceptibility (six items), severity (five items), benefits (three items), barriers (four items), self-efficacy (10 items), health motivation (five items), and cues to action (five items). The items in the seven predictor categories were measured on a 5-point Likert-type scale, namely, 5—strongly possible, 4—possible, 3—neither possible nor impossible, 2—impossible, and 1—strongly impossible. Negative items were scored reversely so that the higher scores indicate higher levels of the item. The scores on each of the seven scales were averaged and used as independent variables in statistical analysis.

### 2.3. Grouping

We divided the patients into two groups. Group A represented patients who visited ophthalmologist due to eye floaters for the first time; group B represented patients who already visited other ophthalmologist due to the same symptoms. Group B was further divided in to subgroup I and subgroup II according to their symptoms of eye floaters. Subgroup I consisted patients with symptoms worse than the last visit, while subgroup II consisted patients in whom symptoms were the same or even less than the last visit. The data collected from the subgroup II (true doctor-shoppers) were compared with the first time visitors (group A). 

### 2.4. Statistical Analyses

The association of independent variables between first time visitors and true doctor-shoppers was assessed using Chi squared and *t*-tests. Subsequently, first time visitors and true doctor-shoppers were treated as binary variable and logistic regression models were built to assess the effects of all independent variables using the Statistical Package for the Social Sciences (Windows version 20.0; SPSS Inc., Chicago, IL, USA).

## 3. Results and Discussion

### 3.1. Results

In this cross-sectional study, 175 consecutive patients presenting primarily a chief complaint of floaters were enrolled. A total of 143 patients (81.7%) completed the questionnaire. Seventy-seven (53.8%) patients claimed that the visit was their first consultation for eye floaters (first time visitors), while other 66 patients (46.2%) had already visited other ophthalmologist (doctor-shoppers). Among these 66 doctor-shoppers, 16 (24.2%) patients claimed that the symptoms of eye floaters worsened as compared to previous visits. That means there were 50 true doctor-shoppers, with same or even less symptoms, seeking consultation for eye floaters more than once. 

The socio-demographic characteristics of the 143 patients and the comparisons of the two groups are shown in Table 1. More than 60% (87/143, 60.8%) of the patients were women, 64.5% (91/141) were at age of 40 or above, 66.4% (95/143) completed college education, and 50.4% (65/129) had a monthly household income of NTD 60000 or more, which is rather close to the median household income (NTD 64142) in Taiwan in 2009. Analysis of refractory status of myopia showed that 83% of the patients (117/141) had myopia and 32.6% (46/141) were high myopia (myopia 6.0 D or more). Logistic regression was used to examine the potential contributions of socio-demographic variables on doctor shopping behavior. There was no significant difference between first time visitors and doctor-shoppers in all socio-demographic variables except gender and low myopia status. Men (*p* = 0.033), comparing to women, and patients with a low myopia status (*p* = 0.035), comparing to patients without myopia, were less likely to consult ophthalmologist without any worse change of eye floaters. These results are summarized in Table 2. Although not statistically significant, patients with moderate and high myopia patients also showed a trend towards less doctor-shopping behaviors. Among the true doctor-shoppers, the main reasons for visiting include: (1) worrying about the condition or distrusting the diagnosis or explanation given by previous doctors (50%); (2) seeking a second opinion in convenience (28%), and (3) receiving advice from family or friends (4%). 

Logistic regression was used to examine the potential contributions of independent variables on doctor shopping behavior using data of the 77 first time visitors and 50 true doctor-shoppers (Table 2). Motivation was the only variable that significantly correlated with doctor-shopping behavior (*p* < 0.001, Exp(B) = 1.379). It means with every unit increase of the motivation difference between true shopper and the first time visitor, the odds that the true shopper’s doctor-shopping behavior will increase by a factor of 1.379—the higher the level of health motivation, the higher the possibility of doctor-shopping. Other main concepts of HBM (susceptibility, severity, benefits, barriers, self-efficacy, and cues to action) did not attribute to doctor-shopping behavior.

**Table 1:** Socio-demographic data.

Variables	Levels	Primary Visitor	Doctor-Shopper	χ^2^ Test	*p* Value
Sex ( N * = 143)	Male	36	20	0.000	0.992
	Female	56	31		
**Age (N = 141)**	<40	34	16	2.590	0.274
	≥40, <60	34	16		
	≥60	22	19		
**Education (N = 143)**	High school	34	14	2.104	0.349
	College	41	23		
	Postgraduate	17	14		
**Family income**	≤60000	40	24	1.605	0.448
(NTD **) (N = 129)	60001–90000	16	14		
	≥90001	24	11		
**Myopia status**	No myopia	14	10	1.719	0.663
**(N = 141)**	≤3.0 D	23	11		
	>3.0 D, ≤6.0 D	26	11		
	>6.0 D	27	19		

Notes: * N: valid data number; ** NTD: new Taiwan dollar.

**Table 2:** Results of the logistic regression model.

Independent Variables	B ^‡^	S.E. ^‡^	Wald ^‡^	Sig. ^‡^	Exp(B) ^‡^
HBM categories (base-1st time visitor)					
Susceptibility	0.019	0.079	0.058	0.809	1.019
Severity	−0.141	0.083	2.930	0.087	0.868
Benefits	0.126	0.130	0.938	0.333	1.134
Barriers	0.052	0.071	0.539	0.463	1.054
Self-efficacy	−0.034	0.042	0.653	0.419	0.966
Cues to action	−0.430	0.231	3.465	0.063	0.651
Motivation	0.321	0.073	19.203	0.000 ***	1.379
Socio-demographic variables					
Sex (base-female)	−1.131	0.530	4.546	0.033 *	0.323
Age	−0.028	0.020	1.909	0.167	0.973
Education (base-high school)					
college	0.199	0.563	0.125	0.724	1.220
postgraduate	1.061	0.772	1.887	0.170	2.888
Knowledge	0.342	0.200	2.929	0.087	1.407
Family income (base- ≤ 60000 NTD)					
60001–90000	−0.148	0.624	0.056	0.813	0.863
>90000	−0.300	0.615	0.238	0.626	0.741
Myopia status (base-no myopia)					
≤3.0 D	−1.688	0.802	4.432	0.035 *	0.185
>3.0 D, ≤6.0 D	−1.303	0.790	2.721	0.099	0.272
>6.0 D	−1.510	0.795	3.609	0.057	0.221

Notes: B ^‡^: Unstandardized coefficient; S.E. ^‡^: Stander error; Wald ^‡^: Wald chi-square statistic. Statistical significance of individual predictors in logistic regression model is tested using the Wald chi-square statistic. Sig. ^‡^: significance. P-value that smaller than 0.05 is significant. Exp(B) ^‡^: log value of unstandardized coefficient. Odds ratio = Exp(B). *: *p* < 0.05, **: *p* < 0.01, ***: *p* < 0.001.

### 3.2. Discussion

In most cases, eye floaters are secondary to degenerative or aging changes in the vitreous body. In 14% of cases, traction forces from the vitreous jelly on the retina will cause a full-thickness retinal tear, and that will be a risk factor to develop retinal detachment, that could be sight-threatening [1]. Patients with likely PVD should be referred to an ophthalmologist for a complete eye examination at the very first time. The floaters may persist for months or years in cases of chronic, uncomplicated PVD and are not a cause for alarm if no recent change in symptom reported as presence of new shower of floaters or subjective visual reduction [1]. Although most persons develop PVD at some point in their lives, most cases are a benign occurrence without any long-term complication. Because the symptoms may persist or subjectively come and go for a period of time, some patients will shop around ophthalmology clinics or departments for a long period. 

According to a cohort study in Taiwan [15], 2.6% of the cohort had ever visited physicians of the same specialty at different facilities on the same day and the percentage of the cohort with such a doctor-switching behavior increased to 23.5% in a 7-day time frame. It is not only the wastage of medical resources but also burden of medical services employees. Maybe the outpatient clinics in Taiwan, even at the tertiary care medical centers, do not require an appointment for consultation, and patients can choose hospitals or clinics for their own convenience without any restriction by the National Health Insurance (NHI). But patients do obtain benefits by concerning second or even third opinions for several reasons. First, group decision making, or a serial of individual decisions, may be less prone to bias than one decision alone. Second, doctor-shopping may increase patient’s welfare through the alleviation of anxiety or other mechanisms [5]. 

A patient’s journey of seeking medical care is influenced by socio-economic and demographic status, perception of susceptibility and severity, benefits/barriers (or costs) of seeking the interaction, as well as the progress and response to self-care of the illness. Additional influences pertain to the availability and accessibility of social support networks, patient’s knowledge, and experience of the illness [16]. So we included the seven major HBM constructors in the current article: susceptibility, severity, benefits, barriers, self-efficacy, cues to action, motivation, and modifying factors (knowledge, socio- demography, and myopic status) that will affect doctor-shopping behavior. 

In previous studies, women and people with lower education degree were significantly associated with frequent attendance in primary care [4,17], while the age is controversial [3,18,19]. In our study, women were more likely to shop around ophthalmologists due to eye floaters, but not age or education degree. Doctor shopping behavior is common in Taiwan [15]. One possible reason that promotes this behavior in Taiwan is its high availability and accessibility of medical care services. Although reimbursement from the NHI is based on a fee-for-service (fee for each item of services) under global budgeting, the fee is not high enough (ranging from 1.5 USD to 15 USD) to be a barrier for people who want to seek more consultations [15]. Also, we did not find the role of family income in frequent attendance as demonstrated in other study [18]. There was a non-significant lesser doctor-shopping behavior trend in patients with moderate and severe myopia (only the low myopia group was statically significant). A possible explanation is that myopic people may pay more attention to correlated information about myopia and have more knowledge about eye floaters, which bring better familiarity and less uncertainty of the symptoms. 

In HBM, perceived threat may be a function of severity and susceptibility, but, in previous studies, they were presented as separate predictors of behavior, so were the other HBM variables. Congruent with this, Harrison, Mullen and Green’s meta-analysis of HBM showed that, although all correlations between HBM and behavior were statistically significant, the effect sizes were small [20]. That means HBM may not correlate with some behavior under several situations or these variables should not be treated as independent ones.

A critical review of HBM in health behavior studies conducted between 1974 and 1984 permitted an overall assessment of the model’s performance [21]. Perceived barriers were the most powerful single predictor across all studies and behaviors. Perceived susceptibility was a stronger predictor of preventive health behavior than sick role behavior. The reverse was true for perceived benefits. Overall, perceived severity was the least powerful predictor; however, this dimension was strongly related to sick role behavior. In brief, perceived susceptibility may be a weak predictor of sick role behavior. 

The HBM has shortcomings in explaining and predicting behavior. Tanner-Smith concluded the HBM is weak for explaining and predicting perceptions of risk (perceived susceptibility and perceived severity) [22]. An explanation could reside with the HBM’s shortcomings in considering “contextual constraints”. Perceived susceptibility and severity may be high, but if one is struggling with issues such as poverty, additional stressors may supersede actions to assure self-health. For example, if one is barely enough to feed him or family, the concern to seek medical screening or treatment may be secondary. 

Also, the HBM does not consider repeated behavior. Perceived risks may influence the first visit but become less thereafter. For example, a perspective changed between women undergoing a pap screening or mammogram for the first time and those who had made these visits routine [22]. Past behavior was found to be the strongest predictor of both behavioral intention and breast self-examination (BSE). The addition of past behavior after the HBM variables led to significant increases in the amounts of variance explained in BSE intentions and BSE behavior [23,24]. 

According to Bandura’s theory, one’s sense of efficacy is the single and the most necessary motivational element, moving individuals to action [25]. Many researches have also shown that self-efficacy is the main determinant of preventive health behaviors [26,27]. But the decision to develop preventive health behavior is based on factors which are less objective than those symptoms or formal diagnoses given by a doctor. So the impact of self-efficacy on sick role behaviors may be influenced by other variables associated with previous experiences in the target behavior. 

HBM is also limited because it is a cognitively based model and does not consider the emotional component of behavior. Witte considered fear an essential part of a health-related behavior, which was defined as a negative emotion accompanied by a high state of arousal [28]. There are also studies added fear to the model that predicts mammography behavior and have found relationships between HBM constructs and fear [29,30]. The authors concluded that fear was significantly predicted by perceived risk, benefits, and self-efficacy; fear, together with barriers, then predicted actual behavior. As in patients with eye floaters, fear of retinal breaks, retinal detachment, and severe vision loss may predict doctor-shopping behavior. 

Illness perceptions (or illness risk representations) are the organized cognitive representations or beliefs that patients have about their illness. Perception on disease varies among patients. Even patients with the same medical condition can hold very disparate views of their illness [31]. Thus the individuals’ ways of reacting to eye floaters might be different and might depend on the perception of the disease, personal explanation, solutions tried, trust placed in medicine, self-construction, and dispersion of dependency [9]. These representations also elicit emotional arousal such as worry or fear, and both representations and emotions guide the decisions to engage in protective behavior [32]. 

The symptom imagery is likely to be a common type of imagery associated with illness risk because symptoms are primary identity features of illness. Moreover, just as symptom experiences are primary motivators of protective behavior, symptom imagery may trigger protective actions [33,34]. In our study, myodesopsia (eye floaters), a symptom that can last for a long time, may elicit the symptom imagery of getting blind that catalyzes the decision-making process for taking protective action. 

Another psychological reason of frequent attendance was, prior to seeing doctors, patients usually expect some improvements of the illness as a result of seeking medical help [35]. Furthermore, in a fast moving society, e.g., Taiwan, a substantial proportion of the population may expect a quick fix of their illness by doctors’ services. Thus, if the illness does not improve as expected, seeking help from another doctor or a different health care provider is likely to be considered. 

There are several limitations of our study, as the sample size was not large enough, self-estimate questionnaire may not reflect the true situation due to memory or interpretation error, and it’s a cross sectional study of a single disease that can only represent partial condition of medical service seeking behavior.

## 4. Conclusions 

With the framework of HBM, we attempted to explain why patients with same or even less symptoms of eye floaters shopped around ophthalmologists. Among the independent variables (perceived susceptibility, perceived severity, perceived benefits, perceived barriers, self-efficacy, cues-to-action, and health motivation), only motivation was related to doctor-shopping behavior. Thus, unlike preventive health behavior, sick role behaviors of patients with eye floaters may be influenced by factors other than variables in HBM. Fear, symptom imagery, and unsatisfied expectation may be factors that motivate the patients with persisting symptoms to keep on doctor-shopping. HBM may not suitable for sick role behaviors study especially focusing on patients with unexplained, minor, but threating symptoms. Further study with qualitative methods, different model, or cohort study may be considered.
